# Low-dose combined oral contraceptive use is associated with lower bone mineral content variation in adolescents over a 1-year period

**DOI:** 10.1186/s12902-015-0012-7

**Published:** 2015-04-03

**Authors:** Talita Poli Biason, Tamara Beres Lederer Goldberg, Cilmery Suemi Kurokawa, Maria Regina Moretto, Altamir Santos Teixeira, Hélio Rubens de Carvalho Nunes

**Affiliations:** Department of Pediatrics, Adolescent Medicine Discipline, Graduate Program in Gynecology, Obstetrics, and Mastology, Botucatu School of Medicine, São Paulo State University (UNESP), São Paulo, Brazil; Clinical and Experimental Pediatrics Research Center, Department of Pediatrics, Botucatu Medical School, São Paulo State University (UNESP), São Paulo, Brazil; Department of Tropical Diseases and Diagnostic Imaging, Botucatu School of Medicine, São Paulo State University (UNESP), São Paulo, Brazil; Statistical Consultant, Botucatu School of Medicine, São Paulo State University (UNESP), São Paulo, Brazil

**Keywords:** Adolescent, Bone density, Bone mineral content, Contraceptives, Osteoporosis

## Abstract

**Background:**

Low-dose combined oral contraceptives (COCs) can interfere with bone mass acquisition during adolescence. This study aimed to evaluate bone mineral density (BMD) and bone mineral content (BMC) in female adolescents taking a standard low-dose COC (ethinylestradiol 20 μg/desogestrel 150 μg) over a 1-year period and to compare their data with those of healthy adolescents from the same age group not taking COCs.

**Methods:**

This was a non-randomized parallel-control study with a 1-year follow-up. Sixty-seven adolescents aged from 12 to 19 years, divided into COC users (n = 41) taking 20 μg ethinylestradiol/150 μg desogestrel and COC non-user controls (n = 26), were evaluated by bone densitometry examinations at baseline and after 12 months. Comparisons between the groups at the study onset were performed using the Mann–Whitney test with the significance level fixed at 5% or p < 0.05. Comparisons between the groups at the study onset and after 12 months were based on variations in the median percentages for bone mass variables.

**Results:**

The COC users presented with low bone mass acquisition in the lumbar spine, and had BMD and BMC median variations of 2.07% and +1.57%, respectively, between the measurements at baseline and 12 months. The control group had median variations of +12.16% and +16.84% for BMD and BMC, respectively, over the same period. The total body BMD and BMC showed similar evolutions during the study in both groups. Statistical significance (p < 0.05) was seen for the BMC percentage variation between COC users and non-users.

**Conclusions:**

Use of a low-dose COC (ethinylestradiol 20 μg/desogestrel 150 μg) was associated with lower bone mass acquisition in adolescents during the study period.

**Trial registration:**

Registry Number, RBR-5h9b3c.

## Background

Osteoporosis is a major public health issue that results in severe consequences for patients and great expense for public health systems through costly efforts to prevent or treat this disease. Although the disease manifests later in life, measures aimed at its prevention must be adopted during infancy and adolescence, because around 90% of the total bone mass is acquired during that time [1-4]. Inadequate bone mass acquisition during infancy and adolescence is one of the main determinants in the occurrence of osteopenia and osteoporosis later in life [1,4,5]. Genetic factors are responsible for 60–80% of the bone mass increment [6,7], while the remaining bone mass is achieved through other factors such as adequate calcium intake, sun exposure, adequate dietary and supplemental vitamin D intake, and regular physical activity [8].

Additional factors such as gonadal hormones, particularly estrogen, play crucial roles in bone mass acquisition during adolescence. Experimental studies have shown that estrogen reduces osteoclast formation (osteoclastogenesis) and activity, thereby decreasing bone reabsorption, mainly through increased apoptosis. Furthermore, estrogen positively affects the formation, differentiation, proliferation, and activity of osteoblasts, which stimulate bone formation [9-11]. A more specific activity of 17β-estradiol was detected in cell culture, as it negatively modulated osteoclasts, either indirectly by suppressing osteoblastic production of various proresorptive paracrine factors such as interleukin-1β, interleukin-6, and tumor necrosis factor-α, or directly through an estrogen receptor (ER)-mediated mechanism on target cells [12,13]. In addition to the indicated mechanisms, estradiol increased the production of osteoprotegerin by osteoblasts through activation of the estrogen receptor ERα. The cytokine osteoprotegerin is considered to be a potent inhibitor of bone resorption [13].

Therefore, the use of combined oral contraceptives (COCs), which alter the physiology of the hypothalamic–pituitary–gonadal axis, and consequently estrogen levels, may interfere with adolescent bone mass acquisition. Because COCs are the most common contraceptive method used by adolescents and young adults in the United States [14], studies have investigated the consequences of COC use on bone mineral density (BMD) in users at these ages [15,16]. However, some of these studies produced results that have remained controversial.

Gai et al. [15] followed 450 adolescents aged between 16 and 18 years who were taking COCs with 35 or 30 μg ethinylestradiol (EE), and observed no significant differences in BMD between users and non-users after 2 years of observation. Pikkarainen et al. [16] analyzed the effects of COCs containing 20–35 μg EE on bone mass in adolescents aged between 12 and 19 years, and found a smaller increase in bone mineral content (BMC) in users taking COC for over 2 years. However, neither of these studies evaluated the effects on the bone mass in these young people when the COC was no longer taken. Furthermore, specific formulations designed for this age group aim to minimize the risks of thromboembolism and other adverse effects associated with COC use. Nevertheless, the available data on the impact of COC use during adolescence are fairly inconclusive, and therefore new studies on subjects within delimited age groups using the same COC formulations are relevant [17].

This study aimed to evaluate BMC and BMD in adolescents using COCs containing 20 μg EE and 150 μg desogestrel and to compare their data with those from adolescents not using these contraceptives.

## Methods

This was a non-randomized parallel-control study. The participants were followed up for 12 months and data were collected between 2010 and 2012. We recruited 67 healthy female volunteers aged between 12 and 19 years, who attended the Adolescent Medicine Outpatient Clinic of Botucatu University Hospital, São Paulo State University (UNESP), São Paulo, Brazil. The volunteers were post-menarche, had regular menstrual cycles, and had no oligomenorrhea/amenorrhea conditions. None of the participants had previously used hormonal contraceptives, and none of them were pregnant before or at the time of the study. All participants were non-smokers and non-drinkers, and did not participate in sporting activities besides the 2-hour physical activity performed once a week during school hours. Of the participants, 41 were medically advised to start using the COC containing 20 μg EE and 150 μg desogestrel when they were enrolled in the study, and thus constituted the test group (avoidance of unintended pregnancy). The control group consisted of 26 adolescents not using any type of hormonal COCs.

Height and body mass index (BMI) were between the 5th and 95th percentiles for their age group, according to the Centers for Disease Control and Prevention criteria [18]. The health-related exclusion criteria adopted for the study were: history of prematurity or low birth weight; prolonged steroid treatment or use of calcium or iron supplements 12 months prior to the study; diabetes mellitus; acute or chronic malnutrition; congenital or acquired bone diseases; gastrointestinal malabsorption; history of nephropathy with or without chronic renal insufficiency; endocrinopathies; early or late puberty; chronic drug consumption; cystic fibrosis; and celiac disease. Other exclusion criteria included use of medications known to negatively affect bone metabolism such as anticonvulsants and antacids with aluminum, solely vegetarian or high-fiber-containing diet, and daily consumption of more than 300 mg of caffeine or more than 500 mL of cola-based soft drinks.

Blood samples were taken from the COC users at 6 months after entering the study for measurement of estradiol levels by chemiluminescent microparticle immunoassay using an ARCHITECT® Estradiol Kit (Abbott Laboratories, Abbott Park, IL) to indirectly determine the effects of EE use. The percent recovery of estradiol in the presence of ethynylestradiol (interferent content) with this method was reported to be 88.6%.

This study was approved by the Research Ethics Committee of the Botucatu School of Medicine, São Paulo, Brazil. All subjects and their families gave written informed consent for participation in the study.

The COC users underwent anthropometric measurements and evaluation of their nutritional and bone mass at baseline and after 12 months to measure the variables to be examined. The control COC non-users were evaluated for the same variables as the COC users at baseline and after 12 months. Appointments were scheduled for each group every 3 months or according to each patient’s needs. Dietary assessment was performed once at baseline through a 3-day food record to verify calcium intake and factors that could interfere with its bioavailability. The participants were weighed using a Filizola electronic scale with 0.1-kg accuracy and measured for height with a wooden height gauge with 0.1-cm accuracy, and underwent a physical examination to detect any potential alterations. A pubertal stage evaluation was performed according to Tanner by two professionals highly experienced in this function [19].

Anamnesis was performed during all visits, so that any problems or inadequacies with the medication use could be recorded. To evaluate skeletal maturation, bone age (BA) was obtained by the Greulich–Pyle method [20]. Bone mass was evaluated by bone densitometry via dual-energy X-ray absorptiometry (DXA) using a Hologic QDR 4500 Discovery A apparatus (Hologic Inc., Bedford, MA). The bone mass evaluation was adjusted using pediatric software; BMC values were expressed in grams and BMD values were expressed in grams per square centimeter. The areas analyzed by DXA were the lumbar spine region, the whole body, and the whole body without the cephalic region as per the International Society for Clinical Densitometry recommendations, which indicate that these regions have the best accuracy for the infancy and adolescence age ranges [21]. The DXA instrument was calibrated by daily scanning of a hydroxyapatite spine phantom. Machine drift was not observed during the study. The coefficient of variation was estimated from repeated measurements (twice) obtained from 30 patients representative of the clinic’s patient population for all regions mentioned (lumbar spine and total body) after each patient had been repositioned before scanning. With the results in hand, CVs of 0.6% and 1.3% were obtained for the lumbar spine and for the whole body, respectively. All evaluations were made by the same blinded, experienced technician, who also performed the densitometry examinations.

### Statistical analysis

We considered the same standard deviation of 2 for both groups, with type I and type II errors equal to 0.05 and 0.20, respectively, and determined that a sample of 12 adolescents per group would be able to detect variation differences higher than 2.29% between the groups.

Age, weight, height, BMI, and BA presented asymmetric distributions. The Mann–Whitney test was used for homogeneity evaluations between the COC non-user (control) and COC user groups when entering the study, with a fixed significance level of 5%.

The percentages of median variation in BMD and BMC were compared between the groups at 12 months after the initial measurements were taken.

## Results

Among the COC users, 35 completed the study and six were excluded because they opted to discontinue COC use. All 26 individuals in the control group completed the study. There were no significant differences between the groups for age, BA, anthropometric variables, and variables obtained by bone densitometry (Table 1) at baseline. The mean calcium ingestion was 563.21 mg/day in both groups, which was lower than the value of 1300 mg/day [22] recommended in the dietary reference intake (DRI) values. The mean menarche age was similar between the groups and also similar to the mean age of 12.2 years reported for the Brazilian population [23]. In the COC group, the median time interval between menarche and starting COC use (gynecological age) was 48 months, and the median serum estradiol level after 6 months of COC use was 10 pg/mL. These values were comparable to those already reported in the literature for this population [24].

**Table 1:** **Control and COC user group characteristics at baseline (median, minimum, and maximum values)**

	**Non COC (n = 26)**			**COC Users (n = 35)**			***P***
	**Median**	**Minimum**	**Maximum**	**Median**	**Minimum**	**Maximum**	
Age (years)	15.63	14.67	16.08	15.75	11.75	19.50	*0.533*
Bone age (years)	16.00	14.00	18.00	16.50	14.00	18.00	*0.604*
Weight (Kg)	51.90	42.60	64.70	52.20	41.00	73.40	*0.839*
Height (m)	1.64	1.51	1.72	1.59	1.49	1.67	*0.101*
BMI (Kg/m2)	20.02	16.69	24.03	20.88	16.63	27.82	*0.233*
Z-score for BMI	−0.08	−1.40	0.94	0.26	−1.93	1.94	*0.255*
BMI (percentile)	47.08	8.07	82.71	60.23	2.66	97.38	*0.279*
Lumbar BMD (g/cm^2^)	0.85	0.76	1.12	0.96	0.77	1.09	*0.228*
Lumbar BMC (g)	46.37	39.90	76.64	49.82	37.70	64.80	*0.330*
Total body BMD (g/cm^2^)	1.00	0.86	1.18	1.00	0.85	1.13	*0.369*
Total body BMC (g)	1783.62	1260.69	2473.26	1831.42	1291.25	2130.32	*0.855*
Subtotal BMD (g/cm^2^)	0.88	0.75	1.04	0.87	0.73	0.95	*0.503*
Subtotal BMC (g)	1320.19	923.07	1860.11	1407.01	945.38	1609.56	*0.903*
Total body fat (g)	15075.80	9539.10	22160.10	16111.70	8504.00	25681.00	*0.976*
Lean weight (g)	33051.00	13852.00	40656.80	36735.60	29604.00	47615.70	*0.016*
Total body fat (%)	30.10	21.10	37.70	29.50	19.60	38.00	*0.637*

Mann–Whitney test, p < 0.05 indicates significant differences.

In the COC group, height did not vary between the measurements taken at baseline and after 12 months. Although the median weight showed a significant increase (p < 0.001) in this group, the Z-scores and BMI percentiles did not differ significantly. The weight, height, and BMI percentage values in the control group did not show significant differences between the measurements taken at baseline and after 12 months.

At the end of the study, the COC users presented with low variations between the initial and final values for lumbar spine BMD and BMC of +2.07% and +1.57%, respectively, while the control group presented with variations of +12.16% (p = 0.056) and +16.84% (p = 0.014), respectively. The total body BMD and BMC values varied by +0.84% and +1.22% in the COC group, respectively, and were considerably lower than the values of +5.28% and +11.34% in the control group, respectively. The subtotal whole body BMD and BMC values showed a similar variation pattern to the total values. Specifically, the subtotal BMD and BMC values varied by 0.56% and 1.18% in the COC group, and 5.28% and 16.04% in the control group, respectively. Table 2 shows the absolute differences in the variations between the groups, with the lumbar spine BMC (15.27%), subtotal whole body BMC (14.86%), and whole body BMC (10.12%) being the most affected values. Statistical significance (p < 0.05) was observed for the variations in BMC between the COC user and non-user groups, but not for the variations in BMD.

**Table 2:** **Comparison of variations in DXA between the control group and COC users at baseline and after 12 months**

**Variable**	**Non COC**			**COC users**			**Difference** ^**(1)**^	***P***
	**Initial**	**Final**	**Variation (%)**	**Initial**	**Final**	**Variation (%)**		
Lumbar BMD (g/cm^2^)	0.854	0.958	12.16	0.959	0.949	2.07	10.09	*0.056*
Lumbar BMC (g)	46.37	53.73	16.84	49.82	49.03	1.57	15.27	*0.014*
Subtotal BMD (g/cm^2^)	0.879	0.903	5.28	0.874	0.869	0.56	4.72	*0.149*
Subtotal BMC (g)	1320.18	1538.46	16.04	1407.01	1414.69	1.18	14.86	*0.033*
Whole body BMD (g/cm^2^)	1.003	1.042	5.28	0.995	0.992	0.84	4.44	*0.149*
Whole body BMC (g)	1783.62	2006.98	11.34	1831.42	1849.58	1.22	10.12	*0.031*

Variation (in %) at the final moment in relation to the initial moment.

Absolute difference between variations^(1)^.

Mann–Whitney test; p < 0.05 indicates significant differences.

## Discussion

Adequate bone accretion during adolescence is a potential protective factor against the development of osteopenia and/or osteoporosis later in life. Studies on the impacts of COC use on bone accretion are important to clarify whether adolescents taking COCs are at greater risk of developing osteopenia and/or osteoporosis upon reaching menopause. We took a different approach to those previously found in the literature, as we studied a group of adolescents rather than young adults, and tried to exclude those factors known to interfere with bone mass acquisition, such as tobacco and alcohol use. The use of a standardized COC formulation was adopted to avoid different EE doses or different progestagens.

After 1 year of taking a low-dose COC (20 μg EE/150 μg desogestrel), the adolescents in the test group presented small variations in BMD and BMC in the lumbar spine region, while those in the control group exhibited higher variations, which translated as the expected bone mass acquisition during this stage of life. The BMC variations between the control and test groups differed significantly (p < 0.05), while the BMD variations did not. These results reinforce the negative impact of COCs on bone mass acquisition, because the adolescent COC users clearly exhibited lower bone mass acquisition in the lumbar spine region, subtotal whole body, and whole body when compared with the adolescents in the control group. Even though no significant BMD differences were observed between the groups, the variations in bone mass acquisition between the groups were noteworthy. For example, the control group gained 5.28% in subtotal BMD while the test group gained 0.56%, and the control group gained 5.28% in whole body BMD while the test group gained 0.84%. Such variations are noteworthy, even though we cannot pinpoint the biological mechanisms that might be related to these variations or suggest their true meaning at this point in time. In any case, our findings corroborate the data reported in other studies showing that use of COCs at dosages of ≤30 μg EE can interfere with BMD and BMC values in adolescents, reducing normal bone accretion during this stage of life [8,16,25,26].

Recently, Cibula et al. [27] found significantly higher lumbar spine BMD values in COC non-users than in COC users, and higher lumbar BMD in those receiving a higher EE dose, in a study performed on 56 adolescents aged between 15 and 19.5 years. In that study, the authors used a single progestagen associated with different EE doses and observed less bone mass acquisition in the group receiving the lower estrogen dose, suggesting that bone mass accrual depends on estrogen levels. Our findings also revealed changes in bone mass acquisition through analyses of spine BMD and BMC, which were greater in COC non-users than in COC users. These findings can be explained by the fact that trabecular bone, which is considered to be the most important component in vertebra formation and one that has intense remodeling capacity, is more susceptible to interventions than other bones and is also highly affected by estrogen actions [26,28,29].

The change in physiological estrogen production caused by hypothalamic–pituitary–gonadal axis blockade, which results from COC use when associated with low EE concentrations, as found in commonly used formulations, seems to play a fundamental role in low bone accretion in adolescents [30]. Elevated estrogen levels during adolescence, which occur in girls who develop normally during puberty, seem to be positively related to increased BMD [31]. However, during COC use, adolescents presented with estradiol levels that were compatible with the early follicular phase of the menstrual cycle, although the method used here for estradiol measurement was influenced by the levels of interferent content, in this case EE. The adolescents did not reach the elevated levels found in the ovulation phase [24,32]. The estradiol concentrations seen in COC users in our study seem to confirm this hypothesis. It is known that healthy adolescents at the end of puberty present with mean estradiol levels of 87 pg/mL in the follicular phase, reaching ovulatory values of 150–350 pg/mL [33]. On the other hand, some authors described that EE exerts a complex inhibitory effect on periosteal apposition, suggesting that this hypothesis is more plausible. Doubts remain and the bone responses are probably the result of multiple factors [34,35]. A study covering a longer period during adolescence may shed light on the lasting effects of COC use on bone accretion. On the other hand, the use of low EE doses later in life has been reported to promote bone mass acquisition in premenopausal or postmenopausal women [36]. Differences in the actions of endogenous and exogenous estrogen have been demonstrated by Coutant et al. [37] who followed up adolescents at different stages of puberty in another clinical situation. The authors showed that endogenous gonadal steroid secretion increased growth hormone sensitivity in peripubertal normal short girls, while exogenous oral estrogen administered as 17β-estradiol (2 mg once daily) produced a relative decrease or no change in growth hormone sensitivity. The authors concluded that sex steroid concentrations may have exceeded the physiological ranges for the corresponding age [37]. A similar hypothesis can be suggested in our study, as we could not distinguish the action of endogenous estrogen from that of exogenous estrogen. We should also remember that the ER-binding affinity using an identical method for EE relative to E2 was indicated as 1.2:1, respectively, which may result in different responses in target tissues [38]. Experimental studies in rats have suggested that bone tissue is less sensitive to estrogen than the uterus and hypothalamic–pituitary–gonadal axis, indicating that low concentrations of estrogen may block the axis, thereby maintaining uterine trophism, but may not be sufficient to maintain or stimulate adequate bone mass acquisition [39].

The meaning of the effects of the progestagenic component in COCs on bone mass is still poorly understood [8,30,40], owing to difficulties in discriminating between the direct effects of the progestagen on bone, and the effects from alterations in estrogen levels induced by hormonal contraception. Studies have shown that progesterone should act together with estrogen in bone accretion, thereby optimizing the peak bone mass acquisition that occurs in adolescence [41]. Therefore, different progestagen components in COCs could hypothetically influence adolescent BMD and BMC values in different ways. Injectable progestagenic contraceptives, such as Depot medroxyprogesterone acetate (DMPA), have a well-documented negative effect on bone mass acquisition [40,42]. Inhibition of the hypothalamic–pituitary–gonadal axis has been reported among the mechanisms of action for DMPA, which results in a state of hypogonadism, as well as DMPA binding to glucocorticoid receptors, with a reduction in osteoblast activity [43].

However, when used in association with estrogen supplementation during adolescence, the bone mass reduction is minimized [44]. Thus, the low bone acquisition associated with COC use in adolescence seems to be caused by low EE doses [45]. However, there is no available evidence in the scientific literature about how progestagens, when present in COC formulations, can affect bone metabolism.

The effects of DMPA administration on bone mass seem to be reversible because normal BMD is reestablished at 2–3 years after its cessation [46].

This study has associated limitations that include the limited period of 1 year of COC use and the lack of BMD evaluation in adolescents with interrupted COC use, which could have indicated whether inappropriate bone mass incorporation associated with COC use can be reverted. Furthermore, we are aware that our results are based on a small number of adolescents using COCs and that they need to be interpreted with caution. Nevertheless, this study permits the inference with some limitations that a reduction in bone accretion occurred during the use of COC in adolescents who had not yet achieved their peak bone mass.

The clinical relevance of ascertaining low bone accretion in adolescent COC users is still under debate. The risk of fractures in COC users during the second decade of life has not been reported to be higher than that in COC non-users [47]. However, there are no current data in the literature showing that the expected maximum bone mass during adolescence can be reverted when the COC is no longer taken, or the outcomes for COC users as they become elderly people. Thus, the future consequences of the low bone mass acquisition in COC users during the critical period of peak bone mass development remain unknown [48,49].

## Conclusions

Considering the importance of hormonal contraception in adolescents for preventing unplanned pregnancies, new studies are required to establish which estrogenic and progestagenic components, and their ideal doses, would be safe and adequate for appropriate bone mass acquisition in this age group to favor complete development of the bone mineral capital, a protective factor against osteopenia and/or osteoporosis in later life.
